# Artificial Intelligence in Dentistry: Past, Present, and Future

**DOI:** 10.7759/cureus.27405

**Published:** 2022-07-28

**Authors:** Paridhi Agrawal, Pradnya Nikhade

**Affiliations:** 1 Department of Conservative Dentistry and Endodontics, Sharad Pawar Dental College and Hospital, Datta Meghe Institute of Medical Sciences University, Wardha, IND

**Keywords:** electronic brain, deep learning artificial intelligence, convolutional neural networks (cnn), ai, cnn, ann, artificial neural network, applications of ai, artificial intelligence in dentistry, artificial intelligence

## Abstract

Artificial intelligence (AI) has remarkably increased its presence and significance in a wide range of sectors, including dentistry. It can mimic the intelligence of humans to undertake complex predictions and decision-making in the healthcare sector, particularly in endodontics. The models of AI, such as convolutional neural networks and/or artificial neural networks, have shown a variety of applications in endodontics, including studying the anatomy of the root canal system, forecasting the viability of stem cells of the dental pulp, measuring working lengths, pinpointing root fractures and periapical lesions and forecasting the success of retreatment procedures. Future applications of this technology were considered in relation to scheduling, patient care, drug-drug interactions, prognostic diagnosis, and robotic endodontic surgery. In endodontics, in terms of disease detection, evaluation, and prediction, AI has demonstrated accuracy and precision. AI can aid in the advancement of endodontic diagnosis and therapy, which can enhance endodontic treatment results. However, before incorporating AI models into routine clinical operations, it is still important to further certify the cost-effectiveness, dependability, and applicability of these models.

## Introduction and background

One of the most fascinating parts of the human body, the brain, has long piqued the interest of scientists and researchers. The scientific world has never really understood how to create a flawless model that mimics the human brain [1]. For many years, scientists have been working tirelessly to advance "artificial intelligence" (AI) [2]. John McCarthy originally introduced this field of applied computer science known as artificial intelligence in 1956 [3]. It is, at times, called machine intelligence [2]. The "fourth industrial revolution," often known as artificial intelligence, employs computer technology to imitate critical thinking, decision-making, and intelligent behavior that is similar to that of humans [3].

In computer science, the study of an intelligent medium, or any machine that understands its surroundings and acts in a way that maximizes its chances of successfully reaching its goals, is referred to as AI research. The word "AI" is used when the computer imitates analytical functions, such as "learning and problem-solving", that humans frequently associate with other human brains [4]. AI techniques have demonstrated excellent capabilities and capacities in recognizing important data patterns, leading to extensive experimentation with them as clinical trial tools, specifically to assist in decision-making for prognosis and projection, as well as each phase of diagnosis and subsequent therapy [4]. AI has been demonstrated to increase accuracy, efficiency, and precision on par with medical experts more quickly and affordably [3].

Our daily lives are already being impacted by it, thanks to various office and practice management software. Siri, Alexa, and other voice command devices are just a few examples of applications that have built intelligent conversational user interfaces for any device, application language, or environment using artificial intelligence [4]. Virtual and physical (that is robotics) AI are both applicable in the field of health care. The mathematical formulae for medication dosage, diagnosis and prognosis, appointment scheduling, drug interactions, electronic health records, and imaging are the main arena of the virtual type. The physical aspect includes rehabilitation, telepresence, robotic support in surgery, and companionable robots for elderly care [3].

The majority of dental applications employ supervised learning, where the training data consists of a large number of samples, each with different characteristics or features (such as pictures of the patient, their sex, age, how many cavities they have, and so on) and determination of ground truth (e.g., whether there was a previous endodontic visit or not) [3]. The biological neuron system with a large number of connections of neurons that are utilized in "learning” is mimicked by artificial neural networks (ANNs) and is used by its algorithm to comprehend the relationship between attributes and the ground truth [3].

By developing solutions to different clinical problems, thereby making physicians' work easier, artificial intelligence has the potential to revolutionize the medical and dental disciplines [3]. Applications of AI in the dental industry are not routine yet. However, the development of these technologies has had an impact on robotic assistance, dental image diagnostics, caries detection, radiography and pathology, and electronic recordkeeping [3]. In line with the expansion of other dental specialties, endodontic AI research has increased. Regarding the use of AI, endodontists' expertise has to be updated [3]. As a result, this review aims to put forth the literature on the applications of Artificial Intelligence in all dental sectors, especially in endodontics, for diagnosis, making clinical decisions and forecasting successful therapy, as well as to find any present limitations in the usage of AI.

## Review

**Figure 1:**
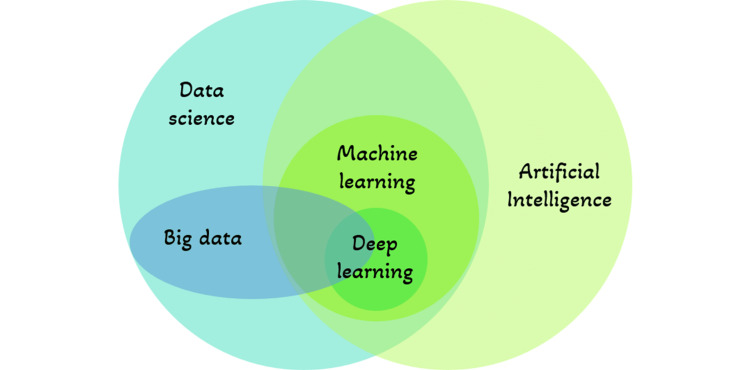
Key elements of Artificial Intelligence Systems. The image is created by one of the authors.

Artificial intelligence, a major invention that imitates human cognitive capabilities, has captured the attention of scientists all around the world [5]. The core component of artificial intelligence technology is a neural network that is designed like that of human brains, which can also simulate human thought. Strongly interconnected neurons make up this type of brain architecture, which primarily functions as a data processing system to address a particular issue [6]. It is a rapidly evolving technology that allows robots to carry out formerly human-only jobs [7]. Recently, it has started to be used in dentistry, which has resulted in exceptional achievements [5]. AI is an efficient method for analyzing clinical dental data [8]. AI developments hint at potential advantages for health care, including fewer postoperative complications, higher quality of life, better decision-making, and far fewer needless procedures [7]. Knowledge of the fundamental components of current artificial intelligence systems in use in society is crucial for having a thorough understanding of AI, as illustrated in Figure 1: AI capacity of a computer to demonstrate its own intellect through the resolution of issues using data [9]. Machine learning: methods used to predict results out of a data set. Making it easier for machines to acquire data already available and resolve problems without human intervention is the goal [9]. Neural networks: use artificial neurons and compute signals which execute similarly to that of the human brain [9]. Deep learning: has numerous computational layers that create a network of neurons that identifies patterns on its own thereby improving detection [5]. Data science: a process of analysis of data and extraction of information from the analyzed data [10]. Big data: analyses a huge amount of data that is steadily expanding in the right direction over years to give consumers correct information [11].

How do artificial intelligence models work?

AI operates in two phases: "training" in the first phase and "testing" in the second. The parameters of the model set are determined by the training data. Retrospectively, the model makes use of data from prior examples, such as patient data or data from data sets containing various examples. These parameters are then applied to the test sets [5].

**Figure 2:**
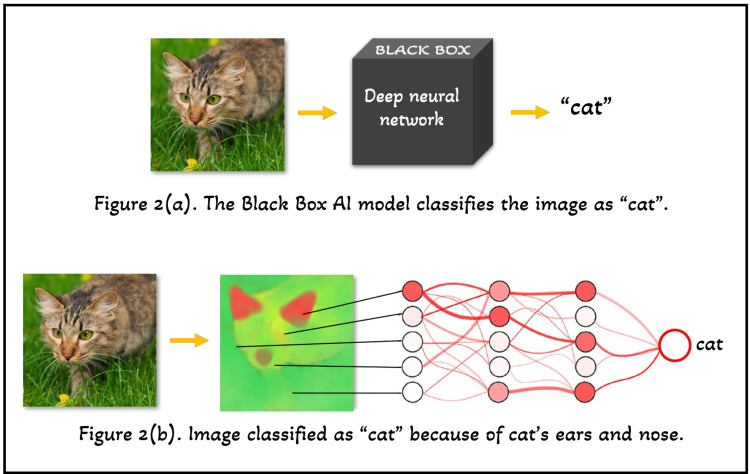
Schematic representation of working of Artificial Intelligence models. (a) Black box AI model. (b) Recent AI models generate heatmaps [12]. The image is created by one of the authors.

Artificial intelligence models were considered “black boxes” because earlier, they provided output without any explanation of why and how they arrived at it (as shown in Figure 2 [a]). On the contrary, today’s AI takes an input (for example, any image as shown in Figure 2 [b]), generates a “heatmap” and provides a prediction (for example, “cat” as shown in Figure 2 [b]). This generated heatmap visualizes which input variables (for example, “pixels” as shown in Figure 2) decided the prediction. This makes it possible to discriminate between safe and relevant prediction techniques, such as categorizing cat photos by focusing on the cat's ears and nose [12].

Hierarchy of artificial intelligence system

**Figure 3:**
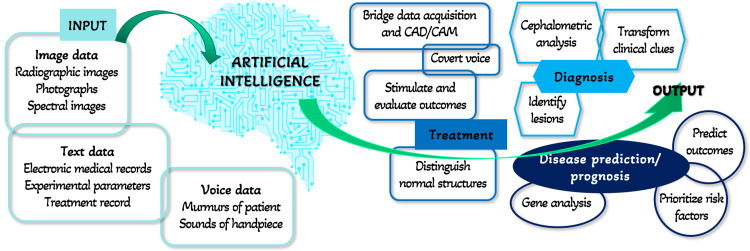
Hierarchy of Artificial Intelligence System The image is created by one of the authors.

As already mentioned, AI, also known as Machine Intelligence, functions like machines. As shown in Figure 3, it adheres to the fundamental machine hierarchy of Input, Processing, and Output [13]. In dentistry, the input data might be voice data (sounds of handpiece), text data (medical or treatment records, experimental parameters), or picture data (spectral or radiographic images, photos). The neural networks process this input data and provide an output. The result might be a prognosis, diagnosis, treatment, or disease prediction. It can interpret clinical cues, do cephalometric analysis, or recognize lesions based on voxel differences to arrive at a diagnosis. It predicts the treatment of the provided input by distinguishing the normal structures, stimulating and evaluating the outcomes, converting the voice data, or bridging data acquisition and CAD/CAM. The artificial intelligence program may anticipate the disease or its prognosis by gene analysis, risk factor prioritization, or outcome prediction.

Applications of artificial intelligence in endodontics

**Figure 4:**
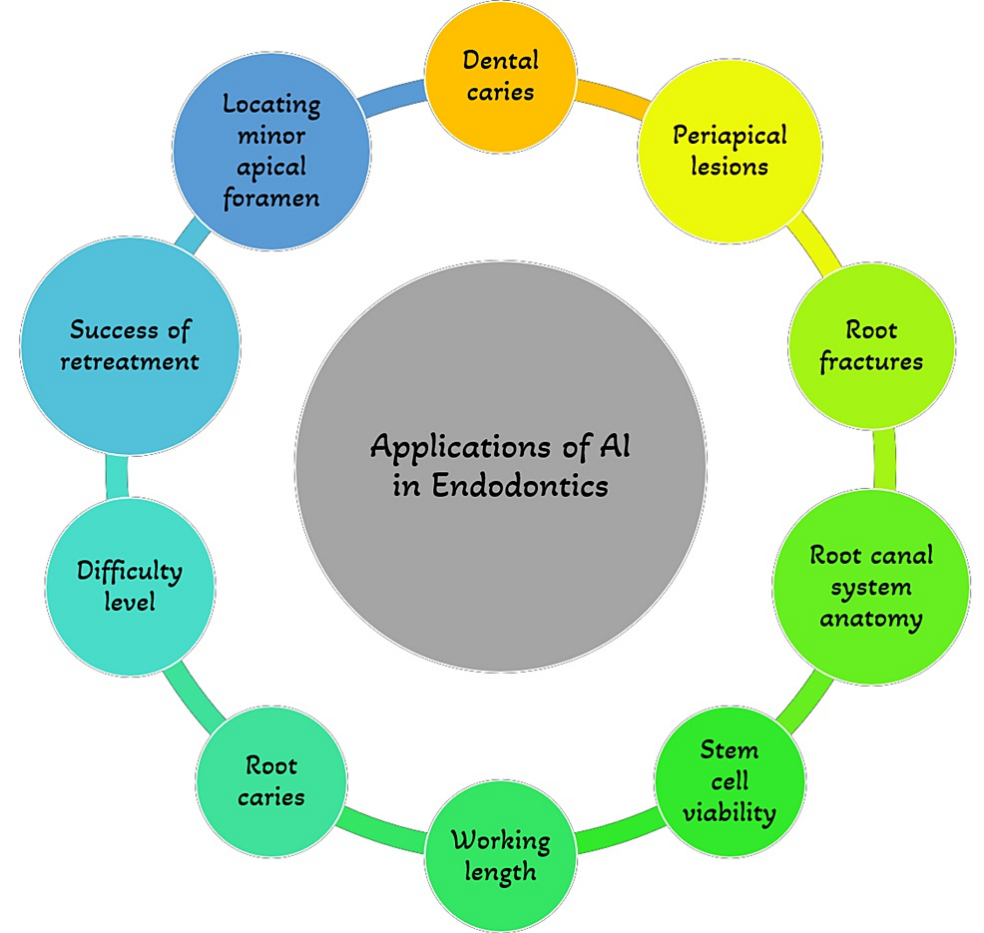
Applications of Artificial Intelligence in Endodontics The image is created by one of the authors.

In endodontics, artificial intelligence is gaining more relevance [14]. Its significance in endodontic treatment planning and disease diagnosis is growing at the moment [15]. Even trivial to minuscule changes at the level of a single pixel that the human eye could miss can be found using AI-based networks [16]. A few of its applications in endodontics are mentioned in Figure 4 and described in detail below:

Periapical Lesions detection

It might be difficult for clinicians to determine a diagnosis and a plan of treatment for teeth showing periapical lesions and/or their symptoms [3]. Approximately 75% of instances with radiolucent jaw lesions are caused by the prevalent condition - apical periodontitis [17]. Early detection may increase the efficacy of treatment, preventing it from spreading to other tissues and reducing potential problems [18]. IOPA and OPG are the two 2-dimensional diagnostic methods that are most frequently employed in everyday clinical practice to detect apical periodontitis [3]. Periapical lesions are often seen as radiolucencies on radiographs. However, because the actual 3-D anatomy is condensed into a 2-D image, the information gleaned from these periapical radiographs is unreliable [19]. CBCT imaging was developed as a 3D imaging technique to precisely detect periapical lesions and assess their location and size [3]. According to a meta-analysis, the periapical lesions' accuracy scores for CBCT imaging, traditional IOPA, and digital IOPA were 0.96, 0.73, and 0.72, respectively [20]. When diagnosing apical periodontitis in teeth with filled roots, CBCT imaging had less accuracy [3].

The characteristics of periapical radiolucency and alveolar bone resorption can both aid in the creation of Artificial Intelligence models for the detection of periapical pathology and periodontitis [21]. Lin et al. suggested two models, first for identifying alveolar bone loss [22] and second for quantifying the extent of the bone loss [23]. Lee et al. [24], based on the level of alveolar bone loss, developed a model formulated on a deep learning network of neurons to identify periodontally challenged molars and premolars and predict hopeless molars and premolars. Mol et al. [25] and Carmody et al. [26] presented models to categorize the severity of periapical lesions with regard to the diagnosis of periapical pathology. According to Endres et al. [27], a deep learning algorithm model can detect periapical radiolucencies on panoramic radiographs as accurately as 24 oral and maxillofacial surgeons. As found by Orhan et al. [28], 142 out of 153 periapical lesions could be detected by the AI system, and this detection accuracy rate was 92.8%. There has been the application of artificial neural networks for the identification of cystic lesions [29]. Additionally, Flores et al. [30] established a methodology to separate granuloma from periapical cysts using CBCT images; it is valued highly in clinical practice because it allows periapical granulomas to recover following root canal therapy without the need for surgery.

Root Fractures Detection

A major outcome that may need root resection or tooth extraction, vertical root fractures (VRF) make up 2% to 5% of crown/root fractures [31,32]. Cone beam computed tomography (CBCT) imaging and radiographs assist in identifying a Vertical Root Fracture which perhaps is challenging to diagnose. And the absence of a conclusive diagnosis might lead to needless surgery or tooth extraction [3]. A clinician's diagnostic options are usually limited by low sensitivity and clinical presentation of traditional radiography in the identification of vertical root fractures.

According to the study by Fukuda et al. [33], CNN may be a useful tool for identifying VRFs on panoramic radiographs. In a different research, periapical radiographs and CBCT images were used to create a neural network to identify VRFs in teeth that were both intact and root-filled [34]. In comparison to pictures from 2-D radiographs, they found that fracture identification of roots on CBCT images is superior in relation to specificity, accuracy, and sensitivity. Shah et al. [35] generated fractures in second molars and used wavelets to analyze them using synthetic data. In a machine learning method, these mathematical operations enable weak signal recovery from noisy settings. Despite a tiny sample size, steerable wavelets were successfully used to detect fractures in high-resolution CBCT images.

Determination of Working Length

Correct determination of WL is crucial for successful root canal treatment outcomes [3]. One method used to assess working length is radiography. Other methods include digital tactile sense, electronic apex locators, the reaction of the patient to a paper point or file point placed into the root canal system, and CBCT imaging [36-39]. Clinical dentists most frequently employ radiography and electronic apex locators as regular techniques. The clarity of the image in digital radiography is essential for the accurate interpretation of the root canal system's anatomy [40]. However, several other factors affect how radiographic interpretations are made, which might lead to misdiagnosis [41]. Consequently, it becomes necessary to use computer-based techniques to provide consistently precise working lengths. According to Saghiri et al. [39], the accuracy of working length assessment can be improved by employing ANNs as a second opinion to locate the radiographic apical foramen. In a different research, Saghiri et al. [42] used a model of a human cadaver to replicate a clinical setting and examined the accuracy of WL assessment by an artificial neural network. When comparing an artificial neural network with the real measurement after extraction, they discovered no change in the root length measurements. Additionally, they noted that when utilizing periapical radiographs to determine minor anatomic constriction, the ANN (96%) outperformed an endodontist (76%) by a wide margin. As a result, an ANN may be thought of as an accurate approach for determining WL.

Morphology of Root and Root Canal System

Understanding the different types of root and root canal systems is a crucial element in the effectiveness of nonsurgical root canal therapy. Cone-beam computed tomography imaging and periapical radiography have often been employed for this purpose. When compared to radiography, Cone beam computed tomography imaging has shown to be more accurate in determining the root and root canal geometries. However, it cannot be advised in standard clinical practice due to radiation problems [3]. According to Hiraiwa et al. [43], the distal roots of the mandibular first molars (radix entomolaris) could be differentiated from one another using a deep learning algorithm that used panoramic radiographs. Lahoud et al. [44] showed an automated, three-dimensional teeth segmentation using the CNN approach. In a rapid, accurate, and effective clinical reference evaluation of 433 cone-beam computed tomographic segmentations of teeth, the authors found that artificial intelligence performed exactly as well as a human operator while working much faster.

Retreatment Predictions

According to the report of Campo et al. [45] for the prediction of the result of nonsurgical retreatment of the root canal with risks and benefits, a case-based reasoning paradigm was designed. In essence, the system advised on whether to retreat or not. The system contained information on statistical probability, performance, and recall. One of the system's strongest aspects is its ability to correctly forecast how the retreatment would turn out. The restriction was that the precision of the system could only match the information in the data [3]. The process of coming up with solutions to issues based on experiences with related issues in the past important knowledge and information may be incorporated by obtaining related situations is case-based reasoning. The problem of variability and the prevalence of various methods might lead to heterogeneity in this system [46]. To increase accuracy, sensitivity, and specificity, future publications should consider the heterogeneity of the human method and possibly increase the sample size [3].

Prediction of the Viability of Stem Cells

A study by Bindal et al. [47] used the neuro-fuzzy inference method and assessed the stem cells extracted from the tooth pulp in many regenerative treatments. By assessing the stem cells' survival following treatment with lipopolysaccharides of bacteria in a model clinical situation, this approach was able to predict the result. To predict cell survival after a variety of regeneration procedures that are subject to microbial infection, the neuro-fuzzy inference system was implied as a tool [47]. The scientists tested the viability of the cells after administering lipopolysaccharide to pulp stem cells to elicit an inflammatory response. The scientists next evaluated the precision of the prediction provided by utilizing adaptive neuro-fuzzy interferences to forecast these stem cells' survival following microbial invasion [3].

Other dental applications of AI

**Figure 5:**
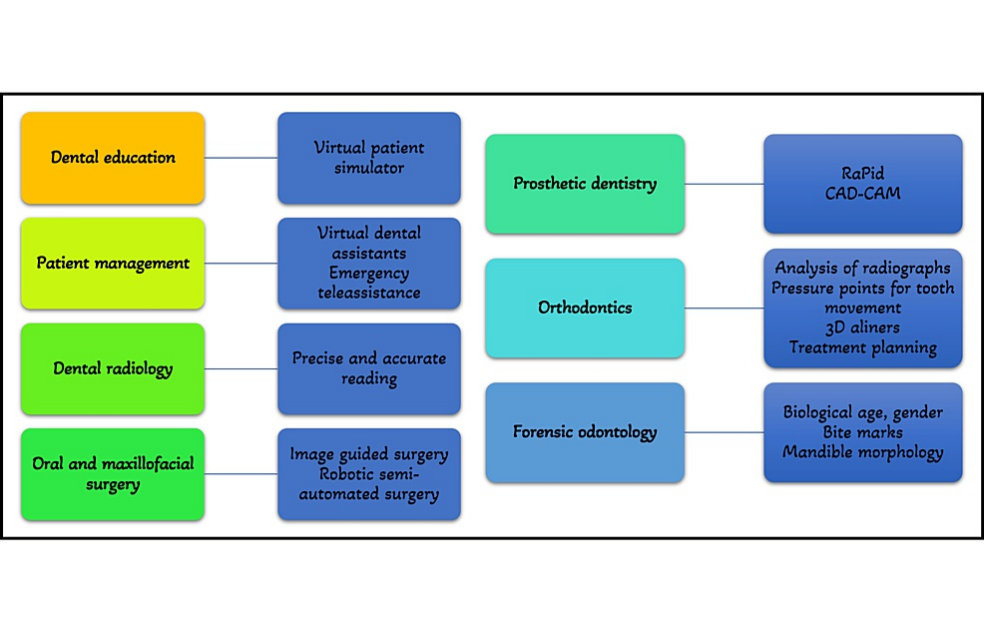
Other applications of Artificial Intelligence in Dentistry

Figure 5 describes other dental applications of AI.

In Dental Education

The area of intelligent tutoring systems has advanced significantly since its start in the 1980s. To generate scenarios that imitate clinical work on patients and minimize all the hazards involved with training on a live patient, AI is frequently employed in the field of dental education. As a result, the preclinical virtual patient feedback to the students has significantly improved. By allowing students to assess their work and compare it to the ideal, the interactive interphase creates high-quality learning settings. Numerous studies on the effectiveness of these systems have shown that students develop a competency-based skill level more quickly with these systems than with conventional simulator units [48].

For Patient Management

Virtual dental assistants powered by artificial intelligence can carry out several duties in the dental office with more accuracy and fewer mistakes, and it requires less manpower for their functioning than an actual human. It can help with clinical diagnosis, treatment planning, scheduling visits, organizing insurance and paperwork, and many more tasks. It is highly helpful in informing the dentist about the patient's medical history and any habits they may have, such as smoking and drinking. In dental emergencies, especially if the practitioner is not available, the patient has the option of emergency teleassistance [1].

For Diagnosis, Treatment, and Prognosis

The application of artificial intelligence in the diagnosis and treatment of oral cavity diseases, as well as in the detection and classification of suspiciously changed mucosa experiencing premalignant and malignant alterations can be beneficial. Even little changes at the single-pixel level that the human eye could miss are picked up. Artificial intelligence may be able to correctly identify a large population's genetic propensity for oral cancer [1]. A useful tool for determining dental prognosis in light of the treatment strategy is an AI-based machine learning system. To determine a tooth's prognosis for long-term oral health and function, a thorough treatment strategy must be carefully reviewed [24].

In Dental Radiology

With more focus on diagnostic procedures in terms of digital RVGS/IOPA, 3D scans, and CBCT, AI is gradually making its way through radiology in dentistry. To create an AI that would aid in quick diagnosis and treatment planning, a lot of data may be acquired and processed [4].

In Oral and Maxillofacial Surgery

The development of robotic surgery, in which human body motion and intellect are replicated, is the biggest use of artificial intelligence in oral surgery. The dental implant, removal of tumors and foreign objects, biopsies, and temporomandibular joint (TMJ) surgery are examples of image-guided cranial surgery procedures that have been successful in clinical settings. Comparative studies of oral implant surgery demonstrate significantly improved accuracy when compared to the freehand procedure, even when performed by competent surgeons. Additionally, there was no discernible difference between experienced surgeons and trainees. Generally, lesser operation time, higher intraoperative accuracy, and safer manipulation around delicate structures have been reported. More comprehensive surgical resection is possible with image guidance, potentially reducing the need for revision surgeries [1]. Surgery has undergone a revolution thanks to AI, and there are now several robotic surgeons who, with growing efficiency, carry out semi-automated surgical procedures under the supervision of a skilled surgeon [48].

In Prosthetic Dentistry

A design assistant called RaPid for application in prosthodontics has combined numerous aspects like anthropological calculations, face dimensions, ethnicity, and patient preferences in order to present the patient with the optimal aesthetic prosthesis. RaPiD links databases, knowledge-based systems, and computer-aided design by using a logic-based depiction as a unifying framework [1]. With advancements in neural networks, laboratories are utilizing AI to autonomously create innovative dental restorations that meet the highest standards for fit, function, and aesthetics. This will benefit dentistry, but it will also have a significant influence on orofacial and craniofacial prosthetics [4].

In Orthodontics

The most talked-about recent invention is personalized orthodontic care powered by AI. Orthodontic diagnosis, planning, and treatment monitoring are now all possible using AI [4]. Analysis of radiographs and images taken by intraoral scanners and cameras can be used for diagnosis and treatment planning. This removes the need for multiple laboratory procedures as well as producing patient impressions, and the findings are often far more precise than human perception [1]. Utilizing accurate 3D scans and virtual models, it is simple to 3D print the aligners according to a unique treatment strategy. As the enormous amounts of data are processed, an algorithm is developed that intelligently determines under what amount of pressure and how patient teeth should be moved, as well as pressure points specific to that tooth or those teeth. The Artificial Intelligence-assisted aligners promise to shorten treatment times and simplify appointment schedules in addition to providing accurate treatment execution and progress monitoring [4].

In Forensic Odontology

AI is a scientific development that has been extensively applied in forensic medicine. It has shown to be quite effective in determining the biological age and gender of the healthy and ill. Additionally, it is employed for analyzing bite marks and predicting mandibular morphology [49].

Dentistry is set to benefit from some of the most fascinating uses of AI. The Dental Chair, a crucial component of the dental practice, saw a significant shift from physiologic, hydraulic pressure chairs, with a manual pump to become electric with attached multiple sensors. The most recent innovation is a voice-command dental chair that doesn't require the doctor to physically do anything. Voice commands are used for all operations. Soon, dental chairs will be able to monitor a patient's vital signs, anxiety level, weight, and the length of the process while also comforting the patient, warning the operating doctors if any variations are found, and so on. This is because all intelligent minds are working tirelessly on AI [4].

Last but not least, one of the most creative uses of AI is in the field of "bioprinting," which allows living tissue and even organs to be created in successive thin layers of cells and may one day be used to reconstruct oral hard and soft tissues that have been lost due to pathological or unintentional causes [48].

Impact of artificial intelligence on dentists

Although there is plenty of talk about how AI can change dentistry, questions remain about whether it will ever completely replace dentists. Dentistry performed by machines and without human interaction does not represent clinical care. Machines cannot provide clinical intuition, intangible perception, or empathy, which are essential to providing individualized healthcare and professionalism. The most fascinating aspect of human-to-human communication cannot be easily translated into computer language [13].

Limitations and future outlook

Despite the promising results of the presented AI models, it is still necessary to verify their generalizability and reliability using appropriate external data obtained from freshly enlisted patients or accumulated from other dental facilities. Future aims of AI research in the dentistry sector include not only raising the performance of AI models to expert levels but also detecting early lesions that are invisible to the human eye [21].

Summary of dental applications of artificial intelligence

AI technologies can help professionals provide their patients with high-quality dental treatment. Dentists may employ AI systems as a supplemental tool to improve the precision of diagnosis, treatment planning, and treatment result prediction. Deep-learning technologies can provide diagnostic assistance to general dentists. Automated technology can speed up clinical processes and boost physician productivity (e.g., automatic completion of electronic dental records by identifying the tooth and numbering). The accuracy of the diagnosis can be increased by using these systems for secondary views [50].

## Conclusions

Artificial intelligence technology has been widely applied in endodontics. According to research on the use of AI in endodontics, the neural networks performed similarly to the dental experts with more accuracy and precision. Artificial intelligence models have also outperformed the specialists in some studies. According to the studies, these applications might be more useful to novices and non-specialists as an expert opinion.

By complementing and, at times, alleviating them, AI should be viewed as an augmentation tool to assist dentists in carrying out more useful tasks, such as integrating patient information and strengthening professional relationships. Contemporary artificial intelligence excels at using structured knowledge and gleaning understanding from vast amounts of data. But it is unable to create associations as the human brain does, and it is only partially capable of making complicated decisions in a clinical situation. In unclear situations, specifically, higher-level comprehension that depends on dentists' experience is necessary to conduct physical examinations, include medical histories, evaluate aesthetic results, and promote conversation. It's critical to stress that good patient-dentist communication requires a nonverbal assessment of the patient's hopes, anxieties, and expectations. This is true despite the contentious debates surrounding the inclusion of empathy into algorithms for affective robots to convey artificial emotions. These communication pathways are intuitive and unplanned.
